# Efficacy of electrical stimulation for treatment of migraine

**DOI:** 10.1097/MD.0000000000017623

**Published:** 2019-11-01

**Authors:** Chao Jiang, Ting Wang, Xiao-yuan Qu, Heng-fang Zhao

**Affiliations:** a The Third Department of Neurology, The Second Affiliated Hospital of Xi’an Medical University, Xi’an; b Department of Emergency, Longhua Hospital Shanghai University of Traditional Chinese Medicine, Shanghai; c School of Economics & Management, Xi Dian University; d Department of Emergency and Intensive Care, Shaanxi Traditional Chinese Medicine Hospital; e Department of Gastroenterology, Xi’an No. 3 Hospital, The Affiliated Hospital of Northwestern University, Xi’an, Shaanxi, P. R. China.

**Keywords:** efficacy, electrical stimulation, migraine, randomized controlled trial, safety

## Abstract

**Background:**

The objective of this study is to assess the efficacy of electrical stimulation (ES) for the treatment of patients with migraine.

**Methods:**

MEDLINE, EMBASE, Cochrane Library, CINAHL, PsycINFO, Scopus, Web of Science, Allied and Complementary Medicine Database, ProQuest Nursing and Allied Health, Chinese Biomedical Literature Database, and China National Knowledge Infrastructure will be searched for eligible studies. All electronic databases will be searched from inception to the present with no language restriction. Two authors will independently carry out study selection, data collection, and study quality assessment, respectively. RevMan 5. 3 software will be used for statistical analysis.

**Results:**

This study will summarize high quality evidence on the efficacy and safety of ES for the treatment of migraine.

**Conclusion:**

This study will establish the accurate results of ES for migraine to facilitate the clinical practice and guideline development.

**PROSPERO registration number:**

PROSPERO CRD42019147480.

## 1 Introduction

Migraine is a very common disorder that occurs repeatedly at the neurological clinic.^[1–3]^ It is often characterized as a pulsating pain on one side of the head, and is often accompanied by nausea and sensitivity to light and sound.^[4–9]^ It has been reported that about 3.3% to 32.6% females and 0.7% to 16.1% males experienced such problem annually.^[10]^ Its mechanisms are complex and still not fully understood^[11–13]^; however, several risk triggers are responsible for such disorder, including hormonal changes in women, drinks, stress, sensory stimuli, sleep changes, physical factors, weather changes, medications, and foods.^[14–17]^ Previous studies have reported that electrical stimulation (ES) has been used to treat patients with migraine.^[18–21]^ However, its results are still not consistent. Therefore, this study will systematically assess the effectiveness and safety of ES for patients with migraine.

## 2 Methods

### 2.1 Criteria for including studies

#### 2.1.1 Types of studies

We will only include randomized controlled trials (RCTs) of ES for patients with migraine. However, we will exclude studies of nonclinical studies, and noncontrolled studies.

#### 2.1.2 Types of interventions

In the experimental group, all participants must receive ES treatment alone.

In the control group, participants receive any kinds of therapies, except all forms of ES.

#### 2.1.3 Types of patients

We will consider RCTs that involved participants who had migraine. There are no limitations on race, gender, and age.

#### 2.1.4 Types of outcome measurements

The primary outcome is migraine pain intensity, as measured by visual analogue scale or other scales. The secondary outcomes include migraine frequency; migraine duration time; migraine-associated symptoms (such as nausea, photophobia); quality of life, as measured by Migraine-Specific Quality of Life Questionnaire or other tools; and adverse events.

### 2.2 Data sources and search

The following electronic databases will be retrieved from inception to the present with no language limitation: MEDLINE, EMBASE, Cochrane Library, CINAHL, PsycINFO, Scopus, Web of Science, Allied and Complementary Medicine Database, ProQuest Nursing and Allied Health, Chinese Biomedical Literature Database, and China National Knowledge Infrastructure. The searching terms include migraine, migraine disorders, headache, migraine attack, and episodic migraine, electrical stimulation, electrical therapy, electrical treatment, randomized controlled trial, controlled study, randomly, blinding, and concealment. The search strategy details for MEDLINE are built in Table 1. We will also adapt identical search strategies to the other electronic databases. In addition, we will also search websites of clinical trial registry, conference proceedings, and reference lists of included studies.

**Table 1:**
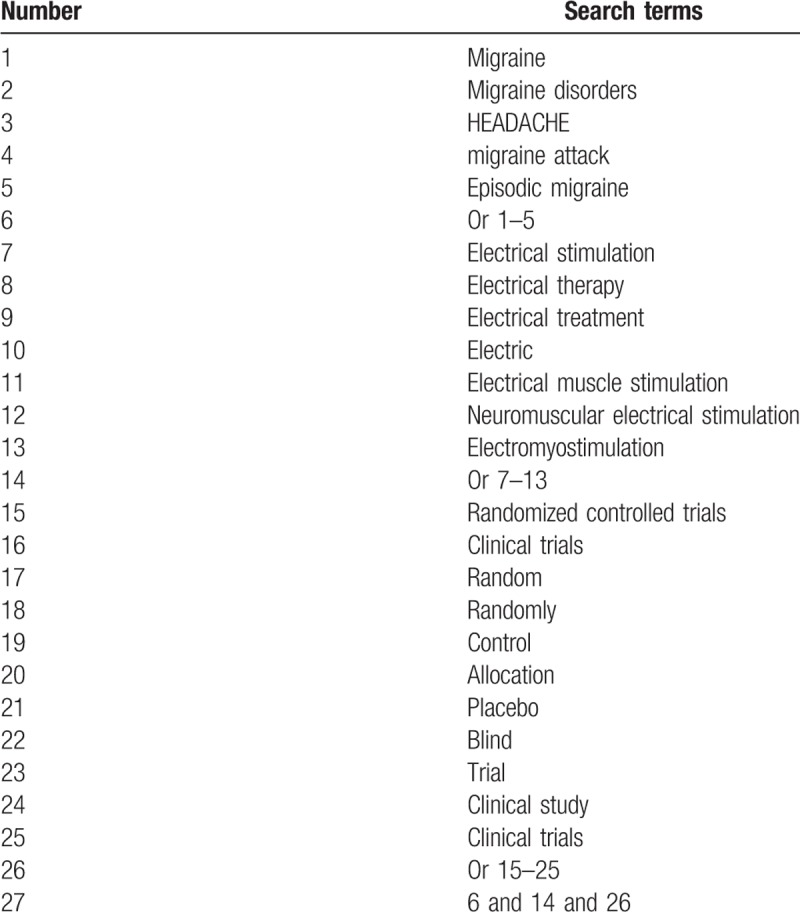
Search strategy for PubMed.

### 2.3 Data collection and analysis

#### 2.3.1 Study selection

We will import all retrieved records into the Endnote X7 and will exclude all duplicated studies by 2 independent authors. After that, they will scan titles and abstracts according to the previous defined eligibility criteria. Then, we will exclude all irrelevant studies, and all remaining studies will be further assessed by reading full texts. Any divergences of study selection between 2 authors will be solved by a 3rd author through discussion or consultation. The process of study selection will be presented in the flowchart with specific reasons for all excluded studies.

#### 2.3.2 Data extraction

Two authors will independently extract information using an advance-designed data collection sheet. The collected information consists of study characteristics (first author, title, published time, etc), patients (gender, age, sample size, disease course, diagnostic criteria, etc), details of interventions and controls (frequency, duration, dosage, etc), outcomes (all primary and secondary outcomes, etc), adverse events, and conflict of interests. Any disagreements between 2 authors will be solved by a 3rd author via discussion.

#### 2.3.3 Missing data dealing with

We will contact original authors for more details on the unclear, insufficient, or missing data. In the absence of reply, we will analyze the available data and will discuss its impacts in the text.

#### 2.3.4 Risk of bias assessment

Two authors will independently assess the risk of bias for all included studies, and any disagreements will be settled by consulting a 3rd author. We will assess the risk of bias for each eligible study based on the Cochrane risk of bias tool. It includes 7 domains, and each filed is further judged as “low risk of bias,” “unclear risk of bias,” and “high risk of bias.”

#### 2.3.5 Subgroup analysis

We will conduct subgroup analysis to address the potential heterogeneity and inconsistency in accordance with the different treatments, comparators, and outcome measurements.

#### 2.3.6 Sensitivity analysis

We will also carry out sensitivity analysis to check the robustness and stability of outcome results by removing studies with high risk of bias.

#### 2.3.7 Reporting bias

We will evaluate the reporting bias via funnel plot and Egger regression test if sufficient eligible studies (normally more than 10 RCTs) are included.^[22,23]^

### 2.4 Data synthesis

In this study, we will apply RevMan 5. 3 software to analyze outcome data and to carry out meta-analysis. All continuous data will be calculated as mean difference or standardized mean difference and 95% confidence intervals, and all dichotomous data will be expressed as risk ratio and 95% confidence intervals. Statistical heterogeneity across included studies will also be evaluated with the *I*^2^ statistics. If the value is *I*^2^ ≤ 50%, the heterogeneity is considered as reasonable, and we will synthesize outcome data with a fixed-effect model. If the value is *I*^2^ > 50%, the heterogeneity is regarded as significant, and we will use a random-effect model for data pooling. If there are at least 2 eligible studies on the same interventions, comparators, and outcome measurements, we will conduct meta-analysis if it is possible.

## 3 Discussion

Currently, ES has been used to manage patients with migraine. Several clinical studies have investigated the comparative efficacy and safety of ES for the treatment of migraine. So far, no study has been carried out to assess the comparative efficacy and acceptability of ES. Thus, it is very necessary to determine the comparative effects of ES.

To our best knowledge, this study firstly explores the efficacy and safety of ES for patients with migraine. On the basis of comparative efficacy evidence and safety, this study is aimed to summarize the most recent evidence of ES in patients who had a migraine. The results of this study may help patients and clinicians choose the best interventions for migraine, as well as the provide evidence for guideline recommendations.

## Acknowledgments

The authors thank the National Natural Science Foundation (8157150927); Important Weak Subjects of Shanghai Health Planning System (Emergency and Critical Disease) (2016ZB0207) for the support. The supporters had no role in this study.

## Author contributions

**Conceptualization:** Chao Jiang, Ting Wang, Xiao-yuan Qu, Heng-fang Zhao.

**Data curation:** Chao Jiang, Ting Wang, Xiao-yuan Qu, Heng-fang Zhao.

**Formal analysis:** Ting Wang, Xiao-yuan Qu, Heng-fang Zhao.

**Investigation:** Xiao-yuan Qu.

**Methodology:** Chao Jiang, Ting Wang, Heng-fang Zhao.

**Project administration:** Xiao-yuan Qu.

**Resources:** Chao Jiang, Ting Wang, Heng-fang Zhao.

**Software:** Chao Jiang, Ting Wang, Heng-fang Zhao.

**Supervision:** Xiao-yuan Qu.

**Validation:** Chao Jiang, Ting Wang, Xiao-yuan Qu, Heng-fang Zhao.

**Visualization:** Chao Jiang, Ting Wang, Xiao-yuan Qu.

**Writing – original draft:** Chao Jiang, Ting Wang, Xiao-yuan Qu, Heng-fang Zhao.

**Writing – review & editing:** Chao Jiang, Ting Wang, Xiao-yuan Qu, Heng-fang Zhao.
